# Strong HIV-1-Specific T Cell Responses in HIV-1-Exposed Uninfected Infants and Neonates Revealed after Regulatory T Cell Removal

**DOI:** 10.1371/journal.pone.0000102

**Published:** 2006-12-20

**Authors:** Fatema A. Legrand, Douglas F. Nixon, Christopher P. Loo, Erika Ono, Joan M. Chapman, Maristela Miyamoto, Ricardo S. Diaz, Amélia M.N. Santos, Regina C.M. Succi, Jacob Abadi, Michael G. Rosenberg, Maria Isabel de Moraes-Pinto, Esper G. Kallas

**Affiliations:** 1 Gladstone Institute of Virology and Immunology, University of California San Francisco, San Francisco, California, United States of America; 2 Division of Experimental Medicine, University of California San Francisco, San Francisco, California, United States of America; 3 Federal University of São Paulo, São Paulo, Brazil; 4 Jacobi Medical Center, Albert Einstein School of Medicine, Bronx, New York, United States of America; New York University School of Medicine, United States of America

## Abstract

**Background:**

*In utero* transmission of HIV-1 occurs on average in only 3%–15% of HIV-1-exposed neonates born to mothers not on antiretroviral drug therapy. Thus, despite potential exposure, the majority of infants remain uninfected. Weak HIV-1-specific T-cell responses have been detected in children exposed to HIV-1, and potentially contribute to protection against infection. We, and others, have recently shown that the removal of CD4^+^CD25^+^ T-regulatory (Treg) cells can reveal strong HIV-1 specific T-cell responses in some HIV-1 infected adults. Here, we hypothesized that Treg cells could suppress HIV-1-specific immune responses in young children.

**Methodology/Principal Findings:**

We studied two cohorts of children. The first group included HIV-1-exposed-uninfected (EU) as well as unexposed (UNEX) neonates. The second group comprised HIV-1-infected and HIV-1-EU children. We quantified the frequency of Treg cells, T-cell activation, and cell-mediated immune responses. We detected high levels of CD4^+^CD25^+^CD127^−^ Treg cells and low levels of CD4^+^ and CD8^+^ T cell activation in the cord blood of the EU neonates. We observed HIV-1-specific T cell immune responses in all of the children exposed to the virus. These T-cell responses were not seen in the cord blood of control HIV-1 unexposed neonates. Moreover, the depletion of CD4^+^CD25^+^ Treg cells from the cord blood of EU newborns strikingly augmented both CD4^+^ and CD8^+^ HIV-1-specific immune responses.

**Conclusions/Significance:**

This study provides new evidence that EU infants can mount strong HIV-1-specific T cell responses, and that *in utero* CD4^+^CD25^+^ T-regulatory cells may be contributing to the lack of vertical transmission by reducing T cell activation.

## Introduction

Vertical transmission of HIV-1 can occur *in utero*, during labor, or after delivery. In the absence of antiretroviral prophylaxis and in a non-breastfeeding population, *in utero* transmission occurs on average in only 3-15% of HIV-1-exposed infants [1]. Multiple factors increase the risk of *in utero* vertical transmission, and include low maternal CD4^+^ lymphocyte count or high viral load, preterm delivery, and chorioamnionitis [2]–[5]. Interestingly, despite potential exposure to HIV-1, including peripartum and through breastfeeding, most infants born to untreated mothers remain uninfected. Several potential genetic, virologic, and immunologic explanations have been provided to explain the lack of transmission (reviewed in [6]), but none have been clearly defined as actively contributing to lower rates of HIV-1 infection in the neonate.

Multiple lines of evidence demonstrate that HIV-1-specific CD8^+^ T cell responses contain and suppress viremia. Such responses are correlated with early control of viral replication during primary infection [7]–[8], and their loss has been linked to rapid disease progression [9]. In the rhesus macaque-simian immunodeficiency virus (SIV) model, the depletion of CD8^+^ T cells leads to increased viral replication [10], which is reversed with the with the reappearance of SIV-specific CD8^+^ T cells [11]. High levels of anti-HIV-1 CD8^+^ T cell responses have been associated with lack of disease in long-term non-progressing patients [9], [12]–[15]. Finally, in both acute and chronic HIV-1 infections, the emergence of CTL escape mutants highlights the role of immunological pressure on viral replication [7], [16]–[17].

HIV-1-specific CD8^+^ T cell-mediated cytolysis is detectable in infected children [18]–[27]; however, these responses are less frequent and weaker in magnitude than in adults and do not always appear before six months of age [28]. Age plays a major role in determining the quality of the HIV-1-specific cellular immune responses. In HIV-1-infected children younger than four years of age, CD8^+^ T cells secreting IFN-γ in response to HIV-1 peptides are low in frequency and lack breadth [27]. Researchers have attributed this to the lack of effective HIV-1-specific CD4^+^ T-helper responses (abnormal skewing of CD4 differentiation to a higher Th2:Th1 ratio) and deficiencies in T-cell priming and effector functions [19], [27]–[29].

HIV-1-specific immune responses have been reported in children who have been exposed to the virus yet remained uninfected. CD8^+^ immune responses to HIV-1 Env, Gag, and Nef proteins have been shown in the peripheral blood of these infants early after birth [19]. In addition, HIV-1-specific CD8^+^ IFN-γ responses have been detected in the peripheral blood of 25% of exposed uninfected infants between 15 to 50 months of age [23] as well as in an uninfected infant using virus-specific stimulation [20]. Recently, it was shown that at one month of age, 58% of infants infected *in utero* and 29% of those infected peripartum exhibited HIV-1-specific cellular immune responses by ELISPOT [28].

The ability of young infants to mount T cell responses has been supported by some recent studies that suggest that neonatal mice are able to generate robust CD8^+^ T cell responses [30]. The responding T cells were able to produce a similar range of cytokines and avidities as that of adults. Following congenital infection, newborns develop a mature CD8^+^ T cell response to human cytomegalovirus (HCMV) [31]–[32]. This response is similar to that detected in adults and the HCMV-specific CD8^+^ T cells express a mature memory phenotype, have antiviral effector functions, produce IFN-γ, and have perforin dependent cytolytic activity (reviewed in [33]). In a study of neonatal cord-blood T cells congenitally infected with *Trypanosoma cruzi*, the protozoan agent of Chagas disease, it was demonstrated that fetal cells have the ability to generate potent and “adult-like” CD8^+^ T cell responses [34].

The role of CD4 T cell help in inducing functional CD8 T cell responses needs further investigation; however, CD4^+^ T cell responses have been enumerated in early life. Young infants are able to mount mature CD4 T cell responses to Bordetella pertussis vaccine [35], to herpes simplex virus infection [36], and to *in utero* exposure to helminth antigens [37]. In HCMV infection, young infants develop mature CD8 T cell responses in the context of low CD4 T cell immune responses whereas in HIV-1 infection CD4 T cell help is integral in controlling HIV-1 infection.

Following the assumption that the young infant can in certain cases mount robust immune responses, we aimed to evaluate the strength of HIV-1-specific immune responses around the time of exposure to the virus and the potential impact of these responses on maternal-infant transmission. We hypothesized that these immune responses may be unmasked upon the removal of CD4^+^CD25^+^ Tregulatory (Treg) cells. We, and others, have shown that this phenomenon occurs in HIV-1 infected adults [38]. We measured HIV-1-specific immune responses and determined the frequency and the suppressive activity of Tregs in the cord blood of HIV-1-exposed uninfected (EU) neonates, as well as in the peripheral blood of HIV-1-infected and EU infants and young children. We detected HIV-1-specific T cell immune responses in all of the children exposed to the virus, including vigorous responses in EU children two years after birth. In addition, depletion of CD4^+^CD25^+^ Treg cells from the cord blood of EU neonates augmented both CD4 and CD8 HIV-1-specific immune responses.

## Materials and Methods

### Patient samples and viral load measurements

HIV-1-infected and control uninfected mothers and their infants were followed and treated at the Federal University of São Paulo hospital (São Paulo, Brazil). Perinatally HIV-1-infected and exposed uninfected pediatric subjects were followed and treated at the Jacobi Medical Center (Bronx, New York). EDTA-treated whole cord blood and peripheral blood samples (2–5 mL) were collected at birth or during scheduled monthly visits after obtaining informed consent based on local Institutional Review Board-approved protocols. An infant was categorized as uninfected if the infant had 2 negative HIV-1 DNA PCR assay results at separate visits 1 month after birth, the second one after 4 months of age. Infants not meeting these criteria were classified as having an indeterminate infection status and were excluded from this study. Positive results of HIV-1 DNA PCR assay were confirmed by HIV-1 RNA PCR assay or HIV culture. Cord blood mononuclear cells (CBMCs) and PBMCs were isolated by Ficoll-Paque PLUS density gradient centrifugation (Amersham Pharmacia Biotech, Uppsala, Sweden) and cryopreserved. Selection bias was avoided by blinding samples for all performed assays. Patient samples were selected on having a cell viability of >80%. Those samples not meeting this criterion were not assayed. All assayed patient samples are represented. Plasma HIV-1 RNA was measured with Amplicor HIV-1 Monitor (version 1.5) with a lower limit of quantification at 400 copies of RNA/mL (Roche Diagnostic Systems, Branchburg, NJ). Absolute levels of CD4^+^ T-cells were determined by flow cytometry with the BD MultiTest CD3/CD4/CD8/CD45 Reagent Kit and analyzed on a FACSCalibur flow cytometer (BD Biosciences, San Jose, CA).

### Immunophenotyping

Cryopreserved CBMCs and PBMCs were thawed, washed, and incubated overnight at 37°C in 5% CO_2_. CBMCs and PBMCs (2×10^5^) were resuspended in PBS (Media Tech, Herndon, VA) and 1% BSA (Sigma, St Louis, MO). CBMCs and PBMCs were stained for activation markers with each of the following monoclonal antibodies (BD Biosciences): CD3-PerCP, CD4-allophycocyanin (APC), HLA-DR-FITC, and QuantiBRITE CD38-PE. CBMCs and PBMCs were also stained for Treg phenotypic markers with each of the following monoclonal antibodies (BD Biosciences): CD3-PE-Cy7, CD4-allophycocyanin-Cy7 (APC-Cy7), CD25-APC, CD127-PE (Beckman Coulter Biosciences), and CD45RA-PE-Cy5. Cells were incubated for 30 min at 4°C, washed, and analyzed on a FACSCalibur flow cytometer, according to the manufacturer's specifications. Calibrated QuantiBRITE fluorescent beads were used to construct a standard curve for quantification of CD38. FlowJo software (TreeStar, Ashland, OR) was employed to convert the measured sample mean fluorescence intensity to antibodies bound per cell (ABC).

### Depletion of CD4^+^CD25^+^ Treg Cells

CD4^+^CD25^+^ Treg cells were purified with MACS CD25 MicroBeads (Miltenyi Biotec, Auburn, CA). Briefly, CBMCs and PBMCs were washed twice in PBS containing 0.5% BSA and 2 mM EDTA, resuspended in PBS containing 0.5% BSA, 2 mM EDTA and 20 µL of MACS CD25 MicroBeads per 10^7^ total CBMC or PBMC, and incubated for 15 minutes at 6 to 12°C. CBMCs and PBMCs were washed twice in PBS containing 0.5% BSA and 2 mM EDTA and applied to a magnetic column on a MiniMACS separation unit (Miltenyi Biotec). CD25-containing and CD25-depleted T-cell fractions were collected. The CD25-containing cell fraction contained 90% CD4^+^ T cells.

### Cytokine flow cytometry

**Figure 1:**
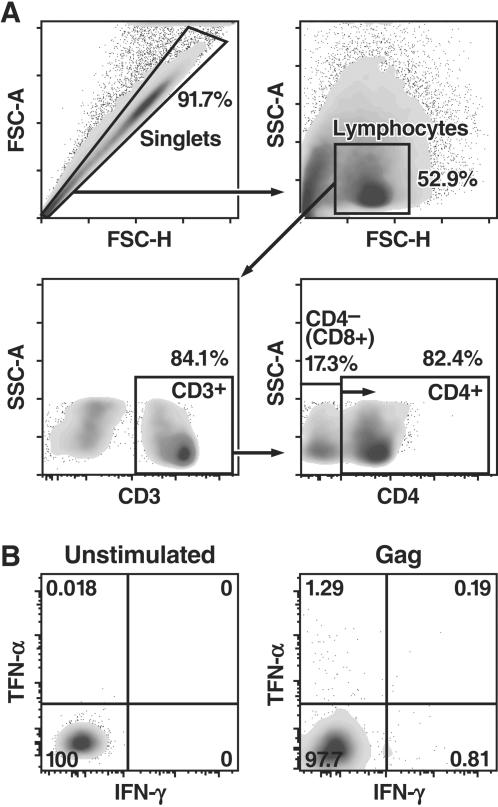
CD4^+^ and CD8^+^ T cell immune responses were measured by cytokine flow cytometry. A) Gating strategy for the identification of polyfunctional IFN-gamma/TNF-alpha CD8^+^ T cell responses. B) Shown are representative data for the unstimulated and HIV-gag-specific response from subject PB-INF-4 after an 18 h *in vitro* stimulation.

Cryopreserved CBMCs and PBMCs were thawed, washed in RPMI-1640 medium (Media Tech) supplemented with 15% FBS (Gemini, Woodland, CA) and incubated overnight at 37°C in 5% CO_2_. HIV-1 specific responses were determined by using pools of overlapping HIV-1 clade B 15-mer peptides spanning the Gag (123 peptides, BD Biosciences) and Nef regions (49 peptides; AIDS Research and Reference Reagent Program, NIAID, NIH). *Staphylococcus* enterotoxin B (SEB, 5 µg/mL; Sigma) served as a positive control antigen. Briefly, 2×10^5^ CBMCs and PBMCs were resuspended in RPMI-15% FBS and incubated with each peptide pool (5 µg/ml for each peptide) at 37°C in 5% CO_2_ for 18 h. Brefeldin A (10 µg/mL; Sigma) was added, and the cells were incubated for an additional 5 h at 37°C in 5% CO_2_. Cells were then washed in PBS with 0.02% EDTA and 1% BSA and transferred to a 96-well V-bottom plate, and permeabilized with a 0.1% Saponin solution, and surface stained with the following monoclonal antibodies (BD Biosciences): CD3-PerCP, CD4-APC-Cy7, IL-2-PE, TNF-α-APC, and IFN-γ-PE-Cy7 for 30 min at room temperature. Finally, cells were washed, fixed with 1% paraformaldehyde, and analyzed on a FACSCanto or LSRII flow cytometer (BD Biosciences). The data were analyzed with FlowJo software. Samples were gated on CD3^+^CD4^+^ or CD3^+^CD4^−^ (CD8^+^) lymphocytes and analyzed for IL-2, TNF-α, and IFN-γ expression (Figure 1). Results were expressed as the percentage of CD3^+^CD4^+^ or CD3^+^CD4^−^ (CD8^+^) expressing IL-2, TNF-α and IFN-γ. HIV-specific responses were considered positive when the response was 2 S.D. above the mean background for all the peptide pools, the cutoff value was 0.13% IFN-γ-producing CD3^+^ T cells.

### Microchimerism Assay

We investigated the presence of maternal cells in each child's circulation using a multiplex PCR for four Short Tandem Repeats Loci (vWA, D8S1179, TPOX, FGA) and a sex-identification marker (Amelogenin locus) discriminated by fragment analysis. The sensitivity of the method includes detection of 1% of mixture of DNAs from different individuals (data not shown). Results showed distinct maternal and child profiles as we were unable to find evidence of cell microchimerism in the children samples.

### Statistical analysis

Statistical analyses were performed with GraphPad Prism (release 4.0, GraphPad Software, San Diego, CA). Comparisons of immune parameters were performed using nonparametric methods, the Mann-Whitney test for independent samples and Wilcoxon matched pairs test for paired samples. Differences were considered significant if p<0.05.

## Results

### Subjects

Our objective was to measure HIV-1-specific T cell immune responses at the time of delivery and within the first year of life. Furthermore, we wanted to establish whether these responses were sustained and whether Treg cells suppressed virus specific responses in an age dependent manner. We evaluated neonatal cord blood samples from a cohort of children in Sao Paulo, Brazil. We performed a retrospective study using cryopreserved cord blood samples from six HIV-1-EU (CB-EU) and four non-exposed neonates (CB-UNEX). All mothers received prenatal care and all HIV-1-infected mothers were placed on triple antiretroviral therapy during pregnancy (Table 1). Five of the 6 mothers had plasma HIV-1 RNA levels below 400 copies/ml in the last month of pregnancy; the sixth mother had a viral load of 3.64 log copies/mL.

**Table 1:**
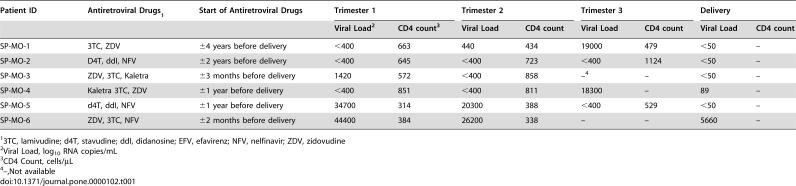
Profiles of HIV-infected pregnant mothers treated at the Federal University of São Paulo hospital (São Paulo, Brazil).

Patient ID	Antiretroviral Drugs^1^	Start of Antiretroviral Drugs	Trimester 1		Trimester 2		Trimester 3		Delivery	
			Viral Load^2^	CD4 count^3^	Viral Load	CD4 count	Viral Load	CD4 count	Viral Load	CD4 count
SP-MO-1	3TC, ZDV	±4 years before delivery	<400	663	440	434	19000	479	<50	–
SP-MO-2	D4T, ddI, NFV	±2 years before delivery	<400	645	<400	723	<400	1124	<50	–
SP-MO-3	ZDV, 3TC, Kaletra	±3 months before delivery	1420	572	<400	858	–4	–	<50	–
SP-MO-4	Kaletra 3TC, ZDV	±1 year before delivery	<400	851	<400	811	18300	–	89	–
SP-MO-5	d4T, ddI, NFV	±1 year before delivery	34700	314	20300	388	<400	529	<50	–
SP-MO-6	ZDV, 3TC, NFV	±2 months before delivery	44400	384	26200	338	–	–	5660	–

1 3TC, lamivudine; d4T, stavudine; ddI, didanosine; EFV, efavirenz; NFV, nelfinavir; ZDV, zidovudine

2 Viral Load, log_10_ RNA copies/mL

3 CD4 Count, cells/µL

4 –,Not available

In addition, we examined peripheral blood samples of young infants within the first year of life as well as a subsequent time-point. These children were followed at the Jacobi Medical Center in the Bronx, NY. We analyzed cryopreserved peripheral blood mononuclear cells from five HIV-1-infected (PB-INF-7mo; median age 7.4 months) and nine EU infants (PB-EU-7mo; median age 6.5 months) as well as five HIV-1-infected (PB-INF-25mo; median age 24.8 months) and seven EU young children (PB-EU-20mo; median age 20 months; Table 2). All HIV-1 infected mothers treated at the Jacobi Medical Center received prenatal care and were on triple antiretroviral therapy during pregnancy. Maternal viral loads were not available for the Jacobi cohort.

**Table 2:**
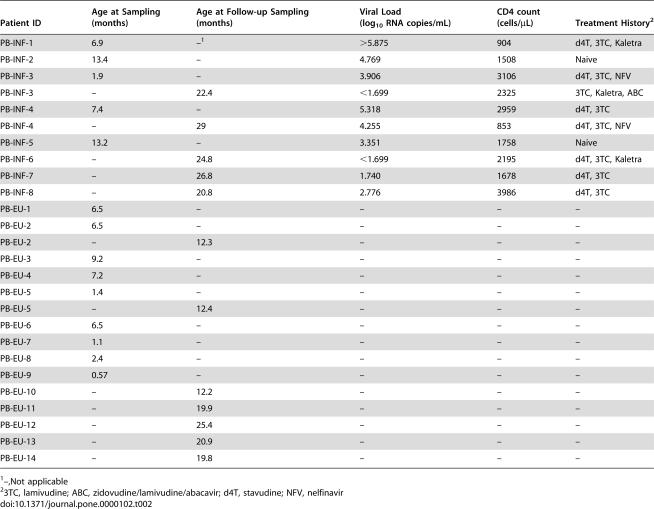
HIV-exposed uninfected infant (median age 6.5 months), follow-up HIV-exposed uninfected young children (median age 19.8 months), HIV-infected infant (median age 7.4 months), and two year follow-up young children (median age 24.8 months) patient characteristics.

Patient ID	Age at Sampling (months)	Age at Follow-up Sampling (months)	Viral Load (log_10_ RNA copies/mL)	CD4 count (cells/µL)	Treatment History^2^
PB-INF-1	6.9	–1	>5.875	904	d4T, 3TC, Kaletra
PB-INF-2	13.4	–	4.769	1508	Naive
PB-INF-3	1.9	–	3.906	3106	d4T, 3TC, NFV
PB-INF-3	–	22.4	<1.699	2325	3TC, Kaletra, ABC
PB-INF-4	7.4	–	5.318	2959	d4T, 3TC
PB-INF-4	–	29	4.255	853	d4T, 3TC, NFV
PB-INF-5	13.2	–	3.351	1758	Naive
PB-INF-6	–	24.8	<1.699	2195	d4T, 3TC, Kaletra
PB-INF-7	–	26.8	1.740	1678	d4T, 3TC
PB-INF-8	–	20.8	2.776	3986	d4T, 3TC
PB-EU-1	6.5	–	–	–	–
PB-EU-2	6.5	–	–	–	–
PB-EU-2	–	12.3	–	–	–
PB-EU-3	9.2	–	–	–	–
PB-EU-4	7.2	–	–	–	–
PB-EU-5	1.4	–	–	–	–
PB-EU-5	–	12.4	–	–	–
PB-EU-6	6.5	–	–	–	–
PB-EU-7	1.1	–	–	–	–
PB-EU-8	2.4	–	–	–	–
PB-EU-9	0.57	–	–	–	–
PB-EU-10	–	12.2	–	–	–
PB-EU-11	–	19.9	–	–	–
PB-EU-12	–	25.4	–	–	–
PB-EU-13	–	20.9	–	–	–
PB-EU-14	–	19.8	–	–	–

1 –,Not applicable

2 3TC, lamivudine; ABC, zidovudine/lamivudine/abacavir; d4T, stavudine; NFV, nelfinavir

### Higher levels of CD4^+^CD25^+^CD127^−^ Treg cells in neonatal cord blood

The current phenotypic definition of Treg cells is based primarily on the expression of CD4 and CD25 as well as the selective expression of the transcription factor Foxp3 [39]–[41]. At the time of this study a human FoxP3 antibody for flow cytometry was not available. However, recent research has demonstrated that CD127 expression is inversely correlated with FoxP3 expression and suppressive function of human Treg cells [42]–[43]. CD127 is part of the heterodimeric IL-7 receptor that plays an important role in the proliferation and differentiation of mature T cells, and *in vitro* experiments show that the expression of CD127 is down-regulated following T cell activation [44]. We therefore quantified the frequency of Treg cells by utilizing a combination of the cell surface molecules CD4, CD25 and CD127. Using flow cytometry, we detected the highest level of CD4^+^CD25^+^CD127^−^ Treg cells in the cord blood of EU neonates (1.55%, Mann-Whitney test; Table 2) as compared to unexposed neonates (0.62%; p = 0.0947) and HIV-1-infected (0.16%; p = 0.0193) and EU infants (0.27%; p = 0.0062).

### Decreased activation levels in HIV-1-exposed-uninfected neonatal cord blood

T-cell activation in HIV-1-infected individuals was measured with the markers HLA-DR and CD38 on CD4^+^ and CD8^+^ T cells. As expected, CD4^+^ and CD8^+^ activation levels were highest in HIV-1-infected infants (Mann-Whitney test, *p* = 0.0732; Table 3). T-cell activation levels as measured by the percentage of cells expressing both HLA-DR and CD38 did not differ significantly in the EU or unexposed neonatal cord blood samples (Mann-Whitney test, *p* = 0.9015 and *p* = 0.8048, respectively).

**Table 3:**
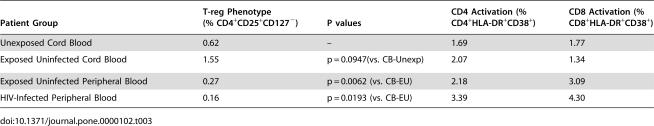
Patient T-regulatory phenotypes (median).

Patient Group	T-reg Phenotype (% CD4^+^CD25^+^CD127^−^)	P values	CD4 Activation (% CD4^+^HLA-DR^+^CD38^+^)	CD8 Activation (% CD8^+^HLA-DR^+^CD38^+^)
Unexposed Cord Blood	0.62	–	1.69	1.77
Exposed Uninfected Cord Blood	1.55	p = 0.0947(vs. CB-Unexp)	2.07	1.34
Exposed Uninfected Peripheral Blood	0.27	p = 0.0062 (vs. CB-EU)	2.18	3.09
HIV-Infected Peripheral Blood	0.16	p = 0.0193 (vs. CB-EU)	3.39	4.30

### HIV-1-specific immune responses in exposed-uninfected neonates and infants

We measured HIV-1-specific as well superantigen-specific immune responses in unexposed and EU neonatal cord blood, HIV-1-EU infants and young children, as well as HIV-1-infected infants and young children. As we were limited by cell number, we chose the most immunogenic proteins Gag and Nef. We selected one HIV structural (Gag) and one non-structural protein (Nef) for our immunological assays. As this was a retrospective study, only two exposed uninfected infants (PB-EU-2 and PB-EU-5) were followed at both an early as well as a late time point. In total, 9 exposed uninfected infants were analyzed at an early time point (7 months of age) and 7 exposed uninfected infants at a later time point (20 months of age). The strength of the CD4^+^ and CD8^+^ T-cell mediated immune response to HIV-1 Gag and Nef proteins was measured by intracellular cytokine production using flow cytometry. We utilized pools of 15-mer overlapping peptides to measure production of IFN-γ in both the cord blood and peripheral blood mononuclear cells.

**Figure 2:**
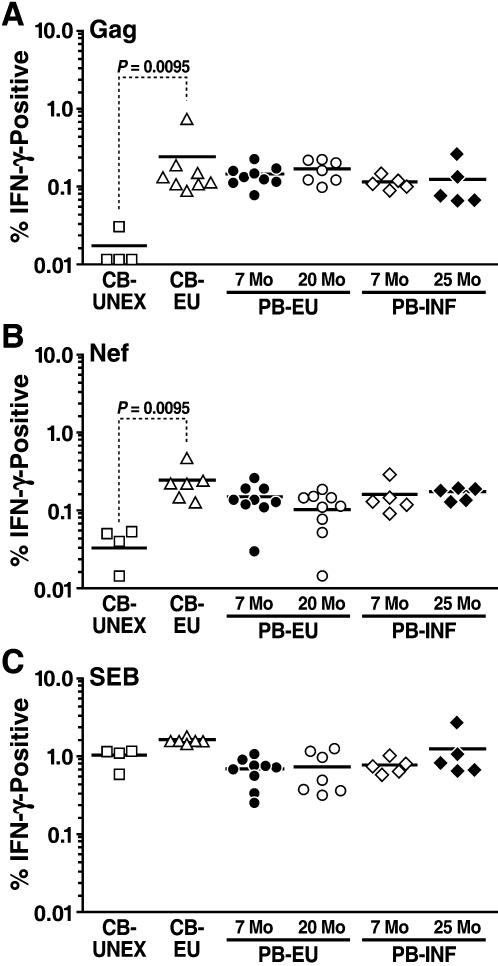
CD8^+^ IFN-gamma T-cell immune responses to HIV-1 Gag *(A)* and Nef *(B)* peptide pools as well as SEB *(C)* in the cord blood of unexposed neonates (CB-UNEX; n = 4), HIV-1-exposed uninfected neonates (CB-EU; n = 6), and in the peripheral blood of HIV-1-exposed-uninfected infants (PB-EU 7 mo; n = 9) and young children (PB-EU 20 mo; n = 7), and in HIV-1-infected infants (PB-INF 7 mo; n = 5) and young children (PB-INF 25 mo; n = 5). Each group is represented by a different symbol.

Significant IFN-γ production by CD8^+^ T cells in response to the Gag and Nef peptide pools was detected in EU neonates, infants and young children as well as infected infants and young children (Figure 2A, 2B). Interestingly, both Gag and Nef specific CD8^+^ T cell IFN-γ responses were sustained in the majority of exposed uninfected children well into the second year of life, and the strength of these responses was comparable to age-matched HIV-1-infected young children. Also, the magnitude of IFN-γ immune responses in EU cord blood was higher than in the peripheral blood. Substantial cytokine production to HIV-1 antigens was not evident in unexposed control cord blood by either CD4^+^ or CD8^+^ T cells (data not shown and Fig. 2, respectively). All groups, including unexposed neonates, elicited a strong CD8^+^ T cell IFN-γ response to *Staphylococcus* enterotoxin B (SEB) (Figure 2C).

### Polyfunctional Gag-specific CD8^+^ T cell immune responses in exposed-uninfected young children

**Figure 3:**
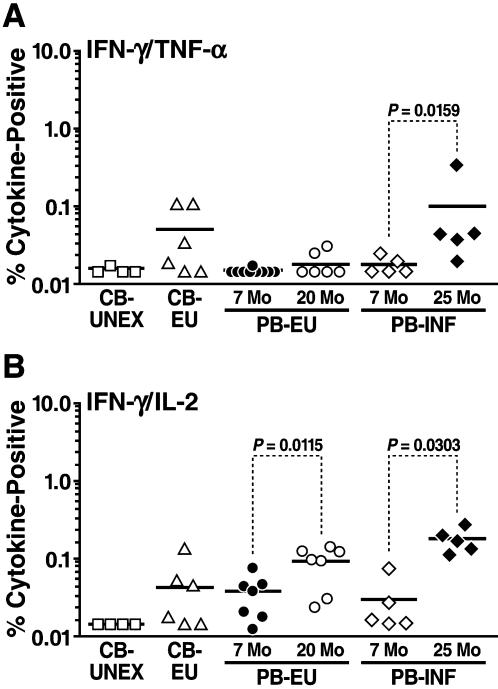
Polyfunctional CD8^+^ T cell immune responses to the HIV-1 Gag peptide pool were detected by cytokine flow cytometry. Responses were measured in the cord blood of unexposed neonates (CB-UNEX; n = 4), HIV-1-exposed uninfected neonates (CB-EU; n = 6), and in the peripheral blood of HIV-1-exposed-uninfected infants (PB-EU 7 mo; n = 9) and young children (PB-EU 20 mo; n = 7), and in HIV-1-infected infants (PB-INF 7 mo; n = 5) and young children (PB-INF 25 mo; n = 5). Each group is represented by a different symbol.

The presence of cells secreting IL-2, IFN-γ and TNF-α has been associated with the lack of disease progression in human long term non-progressors (LTNPs) [45]. HIV-1-specific CD8^+^ T cells that simultaneously produce both IFN-γ/TNF-α upon stimulation have been correlated with cytolytic activity [46]. We detected dual IFN-γ/TNF-α cytokine secretion in the cord blood of EU neonates as well as in HIV-1-infected young children (Figure 3A). IFN-γ/IL-2-secreting CD8^+^ T cells have been shown to promote CD8^+^ T cell proliferation through the secretion of IL-2 even in the absence Ag-specific helper CD4^+^ T cells [47]. These polyfunctional IFN-γ/IL-2 CD8^+^ T-cell immune responses were detected in the cord blood of EU neonates and in the peripheral blood of EU as well as HIV-1 infected infants and children (Figure 3B). IFN-γ/IL-2 CD8^+^ responses were significantly increased in the second year of life in both HIV-1-EU and HIV-1-infected young children. (Wilcoxon matched-pair test*, p* = 0.0115 and *p* = 0.0303, respectively) (Figure 3B).

### Augmented Gag CD8^+^ IFN-γ and CD4^+^ IL-2 immune responses in exposed-uninfected neonatal cord blood upon the removal of Treg cells

**Figure 4:**
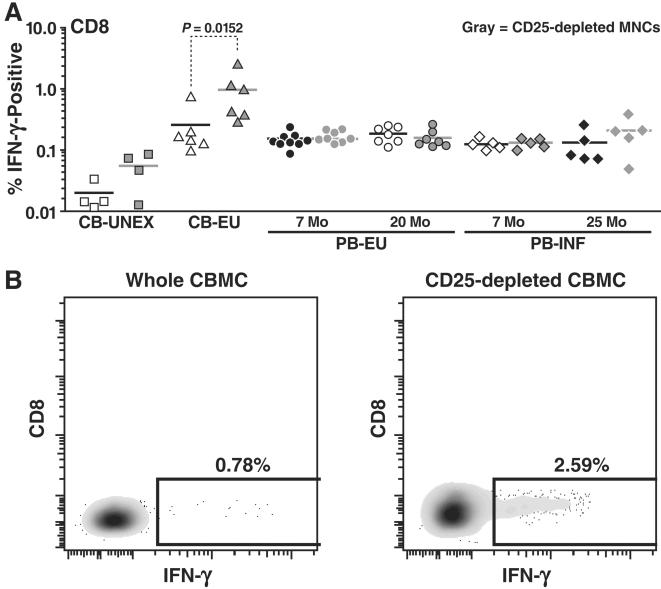
Augmented CD8^+^ HIV-1 immune responses to Gag peptide pools in exposed uninfected neonatal cord blood upon the removal of CD4^+^CD25^+^ Treg cells. A) IFN-gamma production by undepleted whole cord blood and peripheral blood mononuclear cells (MNCs) derived CD8^+^ T-cells (open white symbols) and CD25-depleted MNCs derived CD8^+^ T-cells (closed black symbols) is depicted. B) Flow cytometry plots from an exposed uninfected neonate (Patient 30) representing HIV-1-Gag-induced IFN- gamma production in non-CD25-depleted CBMC derived CD8^+^ T-cells and CD25-depleted CBMC derived CD8^+^ T-cells.

We next wanted to determine the suppressive activity of Treg cells in the cord blood of HIV-1-EU neonates and in the peripheral blood of HIV-1-EU and infected infants and young children. Cells were counted prior to the depletion and then the CD4^+^CD25^+^ Treg cells were depleted. The Treg depleted PBMCs were then recounted and 2×10^5^ cells were assayed for immune responses. The depletion of CD4^+^CD25^+^ T-cells significantly augmented Gag-specific CD8^+^ T cell responses from the cord blood of EU neonates (Figure 4A), increasing by up to 3.37 fold (*p* = 0.0152). In Patient 30, the CD8^+^ T-cell IFN-γ response was particularly robust, reaching 2.59% upon CD4^+^CD25^+^ T-cell depletion (Figure 4B).

**Figure 5:**
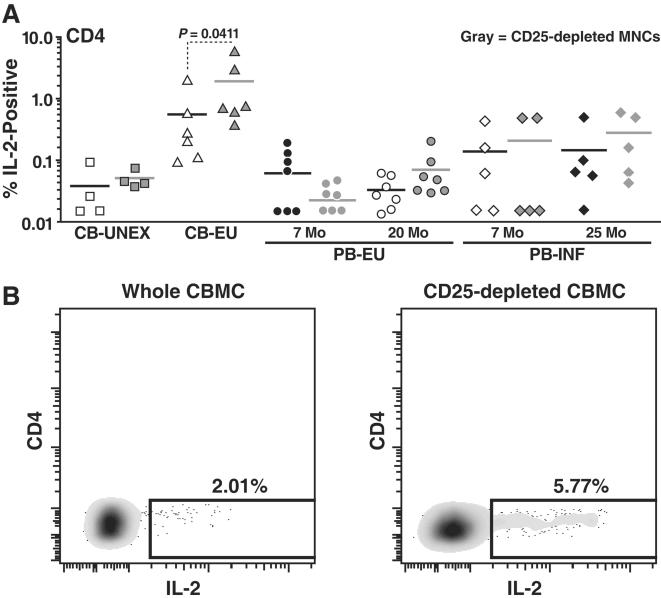
Augmented CD4^+^ HIV-1 immune responses to Gag peptide pools in exposed uninfected neonatal cord blood upon the removal of CD4^+^CD25^+^ Treg cells. A) IL-2 production by undepleted whole cord blood and peripheral blood mononuclear cells (MNCs) derived CD4^+^ T-cells (open white symbols) and CD25-depleted MNCs derived CD4^+^ T-cells (closed black symbols) is depicted. B) Flow cytometry plots from an exposed uninfected neonate (Patient 63) representing HIV-1 Gag induced IL-2 production in undepleted whole CBMC derived CD4^+^ T-cells and CD25-depleted CBMC derived CD4^+^ T-cells.

We also measured CD4^+^ T cell responses to HIV-1 peptides in the presence and absence of Treg cells. Significant IL-2 (Mann-Whitney test, *p* = 0.0158, Figure 5A) cytokine production by CD4^+^ T-cells in response to the Gag peptides were observed in EU neonatal cord blood as compared to unexposed cord blood. Moreover, upon the depletion of CD4^+^CD25^+^ Treg cells, IL-2 responses to Gag were augmented significantly, increasing by up to 2.87 fold (Wilcoxon matched-pair test, *p* = 0.0411). In one EU neonate (Patient 63), CD4^+^ IL-2 production in response to HIV-1 antigens in the whole cord blood mononuclear cell population as well as in CD25-depleted cord blood cells was striking, with responses reaching 5.77% after CD25-depletion (Figure 5B). No significant cytokine production to HIV-1 antigens by CD4^+^ or CD8^+^ T cells was evident in unexposed control whole or CD25-depleted cord blood. In general, both CD8^+^ IFN-γ and CD4^+^ IL-2 immune responses were lower in the peripheral blood than in the cord blood. In the peripheral blood, the depletion of CD4^+^CD25^+^ Treg cells did not significantly impact the magnitude of the CD4^+^ or CD8^+^ T cell immune response in either HIV-1-EU or HIV-1-infected infants and children.

## Discussion

We detected strong, sustained HIV-1-specific immune responses in EU newborns, infants and young children up to two years of age. Unlike previous groups, we assessed HIV-1-specific immune responses in neonates born to women receiving what is now considered appropriate antenatal care for HIV-1-infected pregnant women, which includes antiretroviral therapy to achieve undetectable viral loads before delivery and the provision of elective caesarean section delivery independent of viral load levels [4]. Earlier studies measuring responses from HIV-1 exposed children were conducted prior to the widespread use of HAART in pregnant women as a means to prevent mother-to-child transmission [19]–[20], [22]–[23]. Therefore, it is likely that such children were exposed to high levels of maternal viremia. Our data supports the notion that *in utero* exposure to HIV-1 or its viral products induces HIV-1-specific cell-mediated immune responses even in the setting of low maternal viral loads and does so within a milieu of a low level of immune activation. Moreover, these responses can persist at least well into the second year of life.

The magnitude of these HIV-1-specific memory T cell responses in the absence of productive infection is surprising. It is possible the children were exposed to high quantities of non-infectious HIV-1 particles *in utero*, or alternatively infected maternal lymphocytes or activated antigen presenting cells may have microtransfused across the placenta, stimulating the fetal immune system. However, we did not detect any maternally-derived mononuclear cells in the neonatal cord blood samples. Although the sensitivity of this method is approximately 1%, we cannot preclude the possibility of the presence of maternal cells in compartments other than the blood. Although highly unlikely [48], we also cannot discard the hypothesis that the fetus of an HIV-1-positive mother may become transiently infected with HIV-1 and effectively clears the virus prior to birth. Alternatively, we hypothesize that the prolonged low level of *in utero* antigen exposure during gestation may enable the differentiation of memory cells without a transition through a full effector phase. Perhaps, low levels of antigen at the peak of the response trigger a nominal level of activation allowing for proliferation without the induction of full effector functions, which in turn favor the development of precursors of long-lived memory T-cells.

HIV-1 infection is associated with CD4^+^ T cell loss and progressive immune dysfunction, leading to impaired HIV-1 responses early after infection. It has been hypothesized that the basis for the early decrease in responses to HIV-1 relates to the activity of Treg cells. One theory is that Treg cells influence HIV-1 infection by selectively inducing antigen specific suppression at an early stage of infection, which in turn inhibit the adaptive T cell response against the virus and perhaps self antigens and/or other pathogens. Such suppression of HIV-1 specific T cell responses by Treg cells has been reported in chronically HIV-1-infected adults [38]. Alternatively, it has been proposed that Tregs may help combat HIV-1 disease via suppression of CD4^+^ T cell activation. Oswald-Richter et al. showed that Treg cells are depleted at later stages of HIV infection, which correlated with higher activated T cells, and thus proposed that Treg cells could help reduce viral loads by suppressing T cell activation [49]. This may effectively lower the rate of HIV-1 replication by decreasing the pool of available activated CD4^+^ target cells [49]–[50]. In support, it has been shown that healthy HIV-1-infected individuals have high Treg frequencies [51]. Moreover, patients with Tregs exhibiting strong *ex-vivo* T cell suppression have significantly lower levels of plasma viremia and higher CD4^+^ to CD8^+^ T cell ratios than patients without Treg cell activity [50].

Treg cells have been shown to have an age-dependent loss of suppressive activity, where Treg cell levels are highest early in gestational life and decrease with age [52]. Increased numbers of fetal circulating Treg cells have been shown during early pregnancy, peaking during the second trimester and then declining postpartum [53]. In the cord blood of EU neonates, we observed the highest levels of Treg cells and the lowest levels of T-cell activation. Moreover, CBMCs exhibited full functional capacity including polyfunctional IFN-γ/TNF-α and IFN-γ/IL-2 CD8^+^ immune responses (Figure 3). Similar responses were not detected in infant PBMCs and only emerged in the second year of life. We posit that, *in utero,* Treg cells have a significant role in decreasing activation levels, and in an environment of low activation, polyfunctional HIV-1-specific immune responses, although lower due to Treg activity, are sufficient to subvert vertical transmission. After the neonatal period, in which Treg cells naturally decline, the residual Treg cells in the EU infant may play a minimal role in reducing activation levels and in suppressing immune responses. Only with time, as the infant's immune system matures, do polyfunctional immune responses reemerge. Alternatively, it can be hypothesized that Treg cell suppression of HIV-1-specific T cells, rather than the presence of such responses, may prevent immune activation and susceptibility to HIV transmission.

We measured significantly lower HIV-1-specific immune responses in the peripheral blood than in the neonatal cord blood. This may be a reflection of the fact that these samples are taken later after HIV-1 exposure, wherein the frequency of circulating effector memory cells has naturally waned. It can be further conjectured that antigen presentation by activated and mature maternal cells microtransfused through the placenta may contribute to the activation and augmentation of immune responses *in utero*. Whereas postpartum, within the first year of life, antigen presentation by the infant's immature immune system is incapable of inducing strong polyfunctional immune responses.

Overall, there are a limited number of neonatal immune response studies in human newborns and infants, yet it is believed that qualitative and quantitative differences when compared to adult immune responses exist. The dogma in neonatal immunology has been that newborns have an incompetent immune system, developing only weak or even tolerogenic responses (reviewed in [33]). Multiple lines of evidence, including our own, suggest that this notion needs to be reconsidered. Mature responses to certain vaccines and infectious pathogens have been observed during the postnatal period and even during fetal life (reviewed in [33]). It is evident that a diverse array of cellular immune responses can be developed in early life, and these cells may be able to combat and effectively clear pathogens such as HIV-1. These responses however may be masked or hidden by the presence of antigen-specific suppressor cells such as Treg cells or other as yet unidentified cell populations.

In summary, our data reveals the presence of strong HIV-1-specific T-cell responses in the cord blood and in the peripheral blood of exposed-uninfected neonates and infants, respectively. The magnitude of the immune response is highest in the cord blood and lower in the peripheral blood. What role these cells may play in protection from and/or clearance of vertically transmitted HIV-1 infection is still not known. In the cord blood, CD4^+^CD25^+^ Treg cells significantly reduce activation levels and may provide a mechanism for deterring vertical transmission. Characterization of these HIV-1-specific T-cell responses will have important implications for understanding maternal and fetal immunity against HIV-1 during pregnancy and labor and neonatal vaccine development.
